# The Impact of Different Severities of Anxiety on Decision‐Making

**DOI:** 10.1002/brb3.71704

**Published:** 2026-09-23

**Authors:** Yutong Ma, Yingfeng Tan, Fan Xu, Jiayi Chen, Yuxian Chen, Fangfang Liu, Meng Fan, Qin Han

**Affiliations:** ^1^ College of History and Culture(Tourism) Southwest Minzu University Chengdu Sichuan People's Republic of China; ^2^ Sichuan Provincial Key Laboratory of Philosophy and Social Sciences for Intelligent Medical Care and Elderly Health Management Chengdu Medical College Chengdu Sichuan People's Republic of China; ^3^ Department of Evidence‐Based Medicine and Social Medicine, School of Public Health Chengdu Medical College Sichuan People's Republic of China; ^4^ Department of Pathophysiology, School of Basic Medicine Chengdu Medical College Chengdu Sichuan People's Republic of China

**Keywords:** anxiety state, cognitive modulation, decision‐making, fNIRS, number crunching

## Abstract

**Purpose:**

The effects of anxiety on the brain exist universally; however, little is known about how anxiety affects the functional connectivity of the cerebral cortex and various brain regions. We aimed to understand the relationship between levels of anxiety and decision‐making.

**Method:**

In this study, 58 healthy adult students with an average age of 20 years were recruited. We then estimated their severity of anxiety by using the Self‐Assessment Scale (SAS) for anxiety, grouping them into three severity groups according to their SAS results. Next, we performed a 24‐point number crunching. During this process, the participants' facial expressions and vital signs were also recorded, while functional near‐infrared spectroscopy (fNIRS) was used to measure changes in participants’ hemoglobin in specific cortical areas.

**Finding:**

The moderate‐anxiety group demonstrated superior outcomes compared to the other groups. Specifically, they exhibited more efficient functional connectivity in the brain, smoother hemodynamic changes (primarily reflected by HbO signals, which were selected as the main indicator due to their high sensitivity to task‐evoked cerebral activation), and more positive physiological indicators during the decision‐making task.

**Conclusion:**

The study concludes that a moderate‐anxiety state appears to enhance brain functional connectivity, thereby improving decision‐making ability and accuracy. This research provides valuable insights into the neural mechanisms through which varying severity of anxiety influences cognitive functions like decision‐making.

**Abbreviations**
DBP: diastolic blood pressure
fNIRS: functional near‐infrared spectroscopy
HbO: hemoglobin oxygen
SBP: systolic blood pressure
SDNN: standard deviation of normal‐to‐normal heartbeat
SpO_2_
: peripheral capillary oxygen saturation

## 1 Introduction

Recently, the prevalence of anxiety disorders has garnered significant attention from public health stakeholders. Factors such as social competition, economic uncertainty, and technological advancements have contributed to a notable rise in the occurrence of anxiety disorders. Research on varying levels of self‐reported anxiety in healthy populations has garnered increasing attention. While international studies have focused on anxiety measurement tools, influencing factors, and their impact on individuals' physical and mental health (Y. Wang, Gu et al. 2017), research in China has gradually expanded from clinical populations to healthy individuals. It now emphasizes the manifestations and influencing factors of anxiety within socio‐cultural contexts and increasingly examines the differences in anxiety distribution across various groups (Gomez et al. 2016).

According to a recent report from *The Lancet*, approximately 264 million people worldwide suffer from anxiety disorders (Alonso et al. 2018). Granted, anxiety may be an adaptive response to stress and unpredictable life events; however, depending on its duration and intensity, it can cause serious cognitive deficits. Specifically, anxiety exerts a profound impact on decision‐making (Park and Moghaddam 2017). Undeniably, the impact of anxiety on brain activity is also a significant concern. Previous studies have demonstrated that anxiety substantially alters brain decision‐making processes (Helfinstein et al. 2014), often resulting in decision‐making difficulties and increased threat‐avoidance behaviors (Bishop and Gagne 2018). Research has also shown that emotions play a crucial role in decision‐making, leading to the development of affective choice models that incorporate recent findings on emotional influences (Lerner et al. 2015). The interaction between trait anxiety and facial emotions has been observed during the outcome evaluation stage of decision‐making (Y. Wang, Gu et al. 2017). In recent years, research has focused on cortical regions implicated in anxiety and decision‐making; for example, anxiety‐specific activation in the anterior insula has been linked to interruptions in learning throughout tasks (Nash et al. 2021). Concurrently, decision‐making has been associated with fluctuations in perceptual prediction levels. Negative emotions induced by anxiety tend to reduce decision accuracy (Kraus et al. 2021). Overall, decision‐making is a critical cognitive process that significantly affects quality of life, influencing everything from basic physiological functions to social and occupational performance.

However, most previous experiments have focused on studying simple factors related to the level of anxiety in different populations (Liang et al. 2025; D. Wang et al. 2024). There is a lack of in‐depth research on how varying severities of subclinical anxiety in normal, healthy populations affect decision‐making from the perspectives of neuromodulation and cognitive processes. The precise functional connectivity between the cerebral cortex and the mechanisms underlying its influence requires further investigation.

The impact of varying severities of anxiety on the brain during decision‐making processes is reflected not only in behavior and emotions but also in physiological changes. In recent years, noninvasive techniques such as functional near‐infrared spectroscopy (fNIRS) have been increasingly employed in neuroscience to study brain activity (Wu et al. 2022). By monitoring changes in oxygenated hemoglobin in the brain, researchers can indirectly observe cognitive activities and emotional states. Additionally, vital signs data, including heart rate and respiratory rate, provide valuable insights into the subject's physiological condition. Non‐contact facial expression monitoring is an effective method for recording subjects' emotional expressions (Posse et al. 1996). Furthermore, numerical representation and processing constitute essential cognitive abilities, and mathematical computation is a critical life skill; the underlying thinking processes can effectively reflect the activities and connectivity between brain regions (Hartmann et al. 2020).

In this study, 58 healthy college students with an average age of 20 years were invited to participate in our experiment. They were categorized into three groups based on their Self‐Assessment Scale (SAS) scores for anxiety. The SAS is widely used in clinical and research settings to screen for anxiety disorders and assess the severity of anxiety symptoms (Zung 1971). It demonstrates high sensitivity and specificity in detecting anxiety symptoms (Dunstan and Scott 2020). In our study, SAS scores were standardized according to clinical practice guidelines. Specifically, raw scores were converted into standardized *T*‐scores by multiplying the raw score by 1.25 and rounding to the nearest whole number, as per the original scale's instructions. We applied standard thresholds to interpret SAS scores and categorize anxiety levels: a low‐anxiety group (50 ≤ SAS ≤ 59), a moderate‐anxiety group (60 ≤ SAS ≤ 69), and a high‐anxiety group (SAS ≥ 70). During the experiment, participants engaged in a time‐constrained decision‐making task under induced anxiety. Specifically, they were presented with four numbers on a screen and required to select a sequence of arithmetic operations (addition, subtraction, multiplication, division) to reach the target number 24 within a 60‐s limit. The 24‐point arithmetic task was chosen as a decision‐making paradigm because it engages prefrontal‐parietal regions involved in planning and strategic choice (Hu et al. 2019; Tong et al. 2014). This paradigm operationalizes decision‐making as a process of strategy selection under risk and time pressure, with each selection of an arithmetic operation representing a micro‐decision in a dynamic problem‐solving environment. A camera was positioned in front of the computer to record participants' facial expressions, and fNIRS was used to measure brain activity during the task.

This study aims to investigate how varying severities of self‐reported anxiety (low, moderate, and high) in a healthy population affect decision‐making processes. Specifically, we seek to address the following questions: (1) How do different anxiety levels influence behavioral performance in a numerical calculation task, serving as a proxy for cognitive decision‐making? (2) How do these anxiety levels modulate cerebral cortical activation and functional connectivity, as measured by fNIRS? (3) What corresponding changes occur in vital signs and emotional expressions? By integrating fNIRS, vital signs monitoring, and facial expression analysis, we aim to provide a comprehensive, multimodal understanding of the neurophysiological mechanisms underlying anxiety‐induced alterations in decision‐making.

## 2 Methods

### 2.1 Ethics Statement

This study was pre‐approved by the Ethics Committee of Chengdu Medical College (2023NO.113), and all measurement processes were noninvasive and noncontact. Professional staff monitored the whole process to ensure the smoothness of measurements. All participants provided written informed consent.

### 2.2 Participants

Fifty‐eight healthy college students were recruited to participate in our experiment, including 28 male and 30 female students aged 18–22 years, with a mean age of 20 years. Different groupings were made according to the SAS for anxiety completed by the participants. The low‐anxiety group included 18 participants, the moderate‐anxiety group included 28 participants, and the high‐anxiety group included 12 participants. The inclusion criteria included not being involved with another fNIRS experiment, consent to capture physiological data, and no mental or other psychological diseases. The exclusion criteria included refusal to participate in the study, a history of head trauma or surgery, and prior participation in similar cognitive or decision‐making tasks. Participants with previous exposure to such tasks were excluded to minimize potential confounding effects from learned strategies, practice effects, or familiarity with the task paradigm, which could influence decision‐making performance and fNIRS measurements. Participants were blinded to both their anxiety group classification and the study hypothesis.

### 2.3 General Workflow

Before the start of the experiment, the laboratory environment was quieted down. Upon arrival, participants were instructed to sit and rest for 3 min to stabilize their physiological indicators (Lee et al. 2025). During this resting period, they completed the Self‐Rating Anxiety Scale (SAS) to assess their baseline anxiety levels. The scale instruction asked participants to rate their feelings over the “past week,” so it was used to assess trait anxiety for grouping purposes. All experiments were conducted between 9:00 and 17:00 to control for circadian modulation of anxiety and brain activity (Albrecht et al. 2013; Francis and Porcu 2023). After the resting period, participants were informed of the experimental procedures and precautions. Then, the video equipment and fNIRS device were set up and connected. When the participants were ready, the fNIRS system and video recording equipment worked simultaneously, and the participants followed the instructions of an E‐prime program to engage in the experiment step by step.

**FIGURE 1:**
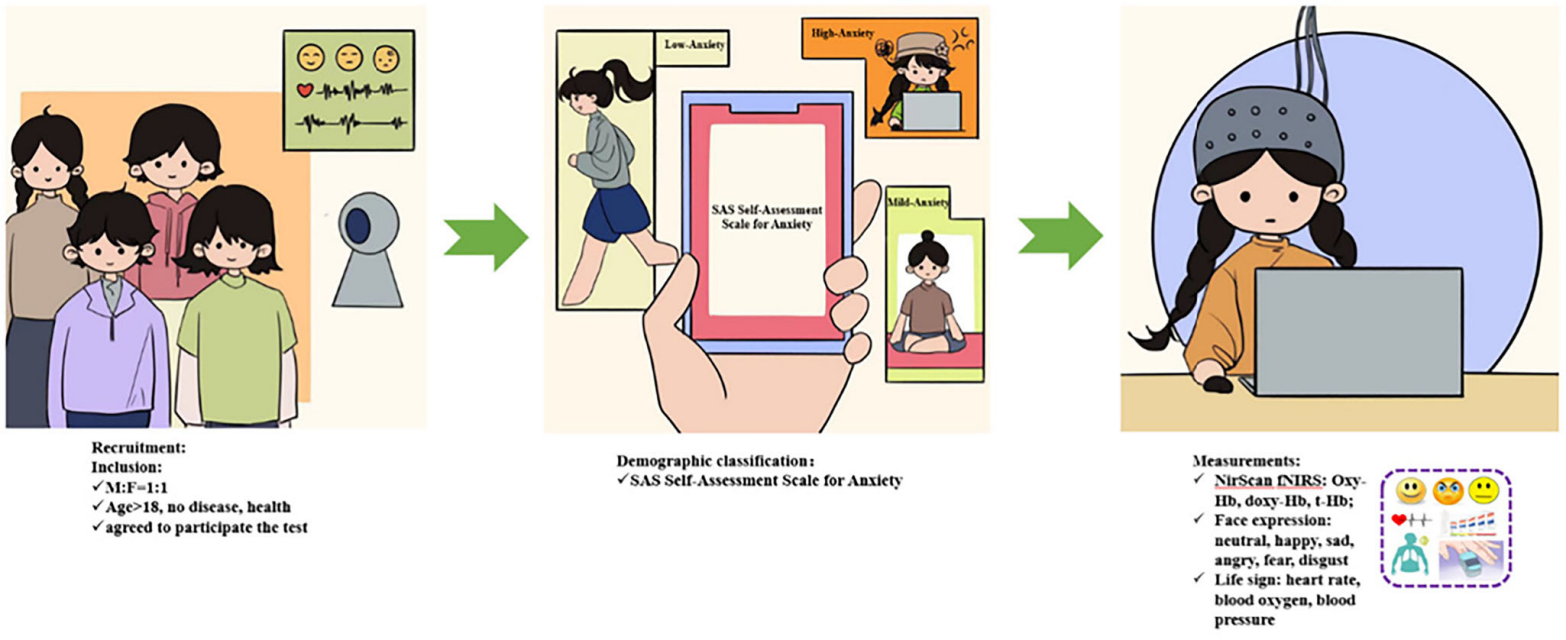
Task demonstration. The study included 58 college students, who were divided into three groups according to their Self‐Assessment Scale (SAS) for anxiety results.

The whole experiment was divided into eight sessions; each session was limited to 1 min in which the participants used any combination of addition, subtraction, multiplication, and division to perform eight different number calculations and obtain a result of 24. In this process, the 1‐min time limit simulated the “stress” of the anxiety situation, and the calculation simulated the implementation of the “decision.” If the participant was not counted within 1 min, the system automatically moved on to the next section of the calculation. During the process, the participants wrote down their calculations during each session on provided paper. Finally, the researchers collected and tallied their data at the end of the experiment to calculate the degree of task completion. Specifically, this task required participants to make rapid computational decisions in a limited amount of time. It coincided with the rapid information processing and evaluation abilities required for decision‐making in anxiety situations. Meanwhile, anxiety may affect participants' attention, working memory, judgment, and decision‐making speed in this context. Consequently, this task was effective in modeling the effects of anxiety on decision‐making efficiency and accuracy. See Figure 1.and Figure.

### 2.4 Measurement of Facial Expressions and Vital Signs

Facial expressions were detected using the face reader Noldus 7.0; meanwhile, Fisheye Vital Signs Monitoring Instrument (Yuwell CM100) was used to measure participants’ vital signs, including heart rate, percutaneous arterial oxygen saturation (SpO_2_), standard deviation normal‐to‐normal heart beat (SDNN), systolic blood pressure (SBP), and diastolic blood pressure (DBP) (Zhang et al. 2024).

### 2.5 fNIRS Data Acquisition

**FIGURE 2:**
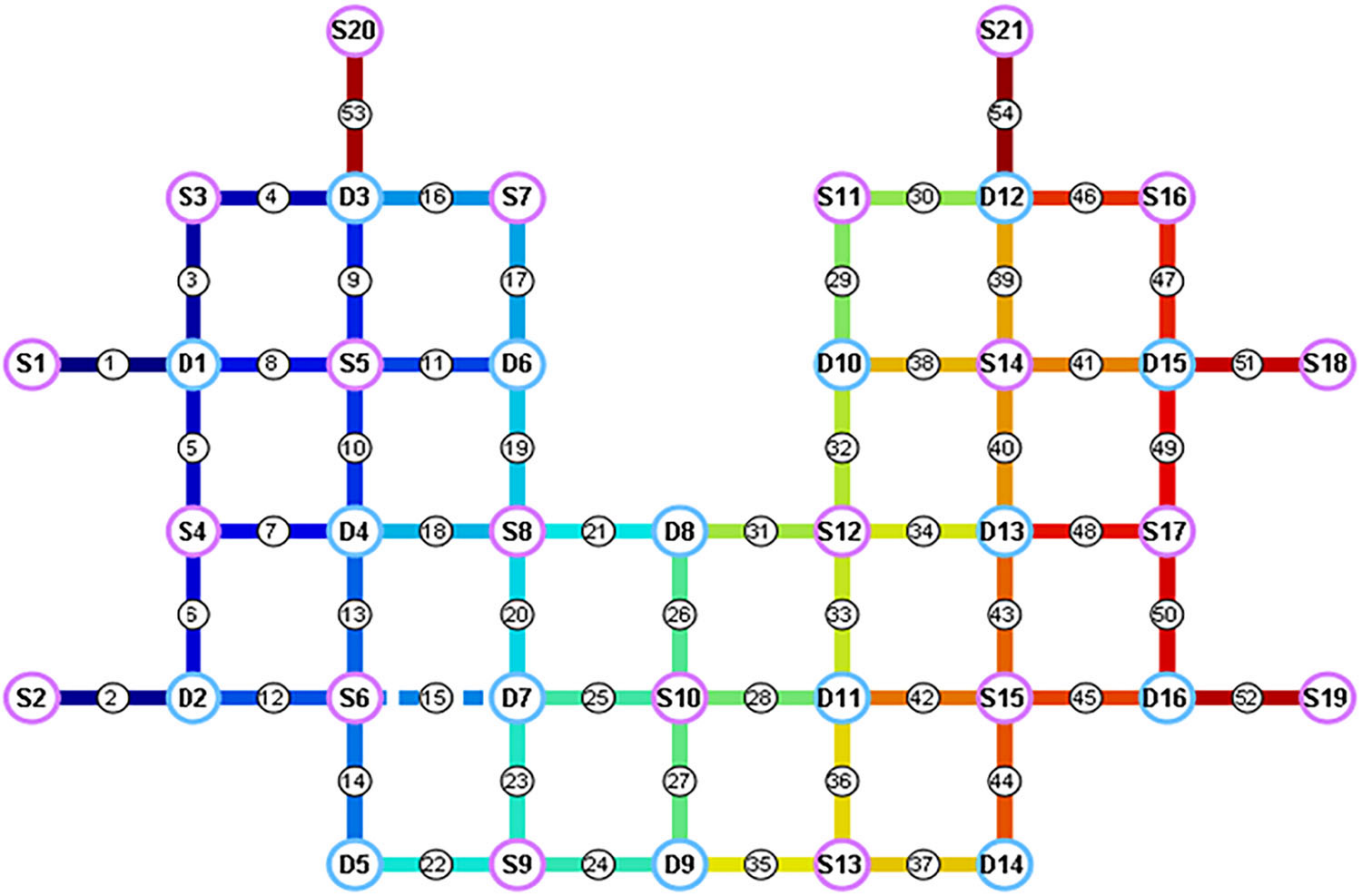
fNIRS schematic layout.

All fNIRS signals were captured by a multichannel fNIRS system (NirSmartII‐3000A, Danyang Huichuang Medical Equipment Co., Ltd., China) with two wavelengths (730 and 850 nm) at a sampling rate of 11 Hz. A stretchable head cap covered the participants’ entire brain area, including the frontal, bilateral temporal, parietal, and occipital lobes. The spatial resolution was approximately 1 cm, and the detection depth sensitivity was about 1.5 cm, which could measure the surface of the cerebral cortex at a depth of 1–3 cm. There were 21 sources and 16 detectors (source–detector separation: 3 cm) on the cap, which formed 54 measurement channels. The spatial locations of sources, detectors, and anchor points (located at Nz, Cz, Al, Ar, and Iz per the standard international 10–20 system of electrode placement) were measured by an electromagnetic 3D digitizer device (Patriot, Polhemus, USA) on the head form (see Figure 2). For the anatomical regions corresponding to each channel and their respective coverage percentages, see Table S1. The acquired coordinates were then transformed into MNI coordinates and further projected to the MNI standard brain template using the spatial registration approach in NirSpace (Danyang Huichuang Medical Equipment Co., Ltd., China) (L. Wang, Ayaz et al. 2017).

### 2.6 FNIRS Data Preprocessing

The fNIRS signals were preprocessed in NirSpark V1.8.1 (Danyang Huichuang Medical Instrument Co., Ltd.). In brief, head motion correction was performed initially, followed by digital bandpass filtering of the raw optical density signals within the 0.01–0.2 Hz range. Second, the relative concentration curves of oxygenated hemoglobin, deoxygenated hemoglobin, and total hemoglobin were obtained using the modified Beer–Lambert law (Mayerhöfer et al. 2020). The path length factor for each wavelength was set to 6, resulting in relative concentration signals for deoxygenated hemoglobin, oxygenated hemoglobin, and total hemoglobin. Finally, we checked the completeness of channels after preprocessing to see if any data were missing, which were then excluded.

### 2.7 Statistical Analysis

All data were stored and managed in Microsoft Office 365, and measurement data were expressed as mean ± standard deviation. Brain functional connectivity was calculated by Pearson correlation coefficients between the time series of the different channels. This constructs the functional connectivity matrix for each subject during the task period. These matrices were subsequently statistically analyzed to assess between‐group differences, including between‐group comparisons, correlation analyses, and brain network visualizations.

Through NieSpark's own statistical plug‐in, a one‐way ANOVA was first used to determine whether there were differences among the three groups, and then multiple comparisons were performed to determine specific between‐group differences. The fNIRS data in the two groups were also compared via a Student's *t*‐test. Stata 18.0 software was used for statistical analysis, while the vital signs and facial expression data were compared via a two‐tailed Student's *t*‐test if the data were normally distributed; otherwise, the nonparametric methods were used. The visualization of vital signs and facial expression analyses were performed by GraphPad Prism 10.03. A *p* value < 0.05 was deemed statistically significant.

## 3 Results

### 3.1 Demographics

Fifty‐eight college students participated in our experimental study. The low‐anxiety group included 18 participants with an average age of 19 years, including 10 males (55.55%) and 8 females (44.45%); their final mathematical accuracy percentage was 55.15%. The distribution of SAS scores is presented in Table 1. Based on the prespecified grouping variable, 18 participants (31.0%) were classified into the low‐anxiety group, 28 (48.3%) into the moderate‐anxiety group, and 12 (20.7%) into the high‐anxiety group. In the overall sample, the mean raw SAS score was 52.83 ± 10.58, with a median of 50.0 and a range of 40–81. The corresponding mean standardized SAS score was 65.72 ± 13.20, with a median of 62.0 and a range of 50–101. The mean standardized scores were 53.78 ± 3.02, 63.96 ± 3.18, and 87.75 ± 9.66 in the low‐, moderate‐, and high‐anxiety groups, respectively. The moderate‐anxiety group consisted of 28 participants with an average age of 20 years, including 12 males (42.86%) and 16 females (57.14%); their final accuracy percentage was 57.05%. The high‐anxiety group had 12 participants with an average age of 20 years, including 6 males (50.00%) and 6 females (50.00%); their final accuracy percentage was 46.67%. Results from *t*‐tests and chi‐square tests revealed no significant differences in age or sex distribution among the three groups, while computation accuracy differed significantly between groups. See Table 2.

**TABLE 1:** Distribution of raw and standardized SAS scores according to anxiety group.

Anxiety group	*n* (%)	Raw SAS score, mean ± SD	Median (IQR)	Range	Standardized SAS score, mean ± SD	Median (IQR)	Range
Low anxiety	18 (31.0%)	43.28 ± 2.54	44.0 (41.0–45.0)	40–47	53.78 ± 3.02	54.5 (51.0–56.0)	50–58
Moderate anxiety	28 (48.3%)	51.43 ±2.70	52.0 (48.5–54.0)	48–55	63.96 ± 3.18	65.0 (60.5–67.0)	60–68
High anxiety	12 (20.7%)	70.42 ±7.80	69.0 (65.0–79.0)	57–81	87.75 ± 9.66	86.0 (81.0–98.0)	71–101
Total	58 (100%)	52.83 ±10.58	50.0 (45.0–55.0)	40–81	65.72 ± 13.20	62.0 (56.0–68.0)	50–101

*Note*: Participants were classified into low‐, moderate‐, and high‐anxiety groups according to their standardized SAS scores. IQR, interquartile range; SAS, Self‐Rating Anxiety Scale.

**TABLE 2:** Demographic information of the three groups.

Vars	Low‐anxiety group (*n* = 18)		Moderate‐anxiety group (*n* = 28)		High‐anxiety group (*n* = 12)		*t/F/χ*2	*p*
	Category/mean	Frequency/SD	Category/mean	Frequency/SD	Category/mean	Frequency/SD		
Age	19.37	0.88	20.01	0.85	20.08	0.81	−0.72	0.48
Sex	Male	10 (55.55%)	Male	12 (42.86%)	Male	6 (50.00%)	0.34	0.56
	Female	8 (45.45%)	Female	16 (57.14%)	Female	6 (50.00%)		
Total ACC	55.15%		57.05%		46.67%		21.95	0.04

### 3.2 Inter‐Group Analysis of Differential Functional Connectivity

**FIGURE 3:**
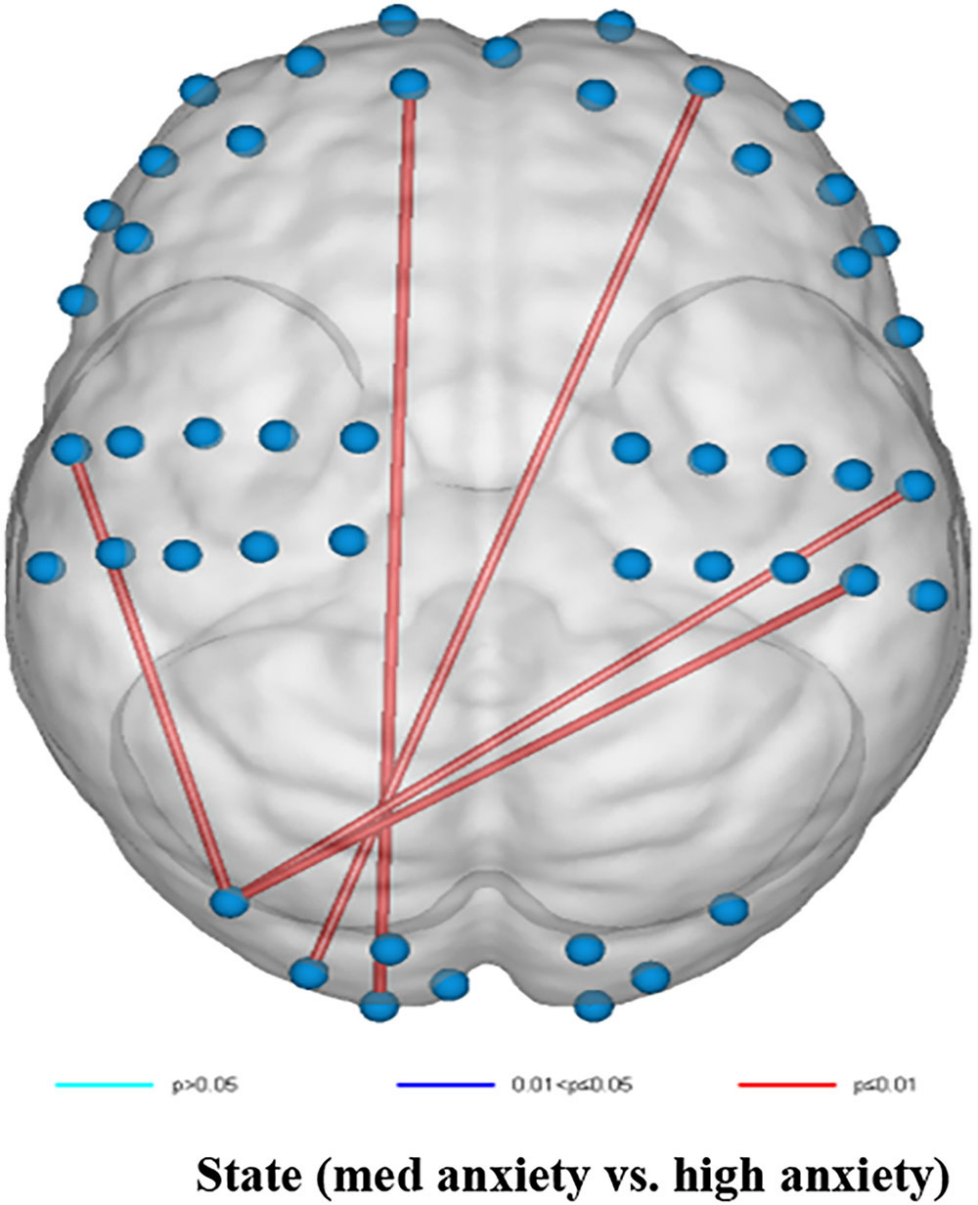
Visualization of functionally differentiated connections. Red lines indicate statistically significant.

A two‐tailed Student's *t*‐test was used to compare the differences in brain functional connectivity strength between the two groups. The results revealed statistically significant differences between the moderate‐ and high‐anxiety groups across five channels (*p* ≤ 0.01). However, these differences were excluded after FDR correction, probably due to a limited sample size. See Table 3, Figure 3, and Table S1.

**TABLE 3:** Results of differences in functional connectivity strength between states (moderate anxiety vs. high anxiety).

CH	Moderate mean	High mean	Moderate std.	High std.	*p*	*T*
1–39	0.2238	0.5347	0.3038	0.2565	0.0037	3.0965
7–40	0.1193	0.4676	0.3354	0.2456	0.0025	3.2350
22–41	0.3358	0.0062	0.3129	0.4699	0.0099	2.7136
26–39	0.1618	0.4297	0.2788	0.2821	0.0085	2.7746
35–39	0.1534	0.5029	0.3423	0.3113	0.0043	3.0364

### 3.3 Visualization of Functional Connectivity During the Task Period in Two Groups

**FIGURE 4:**
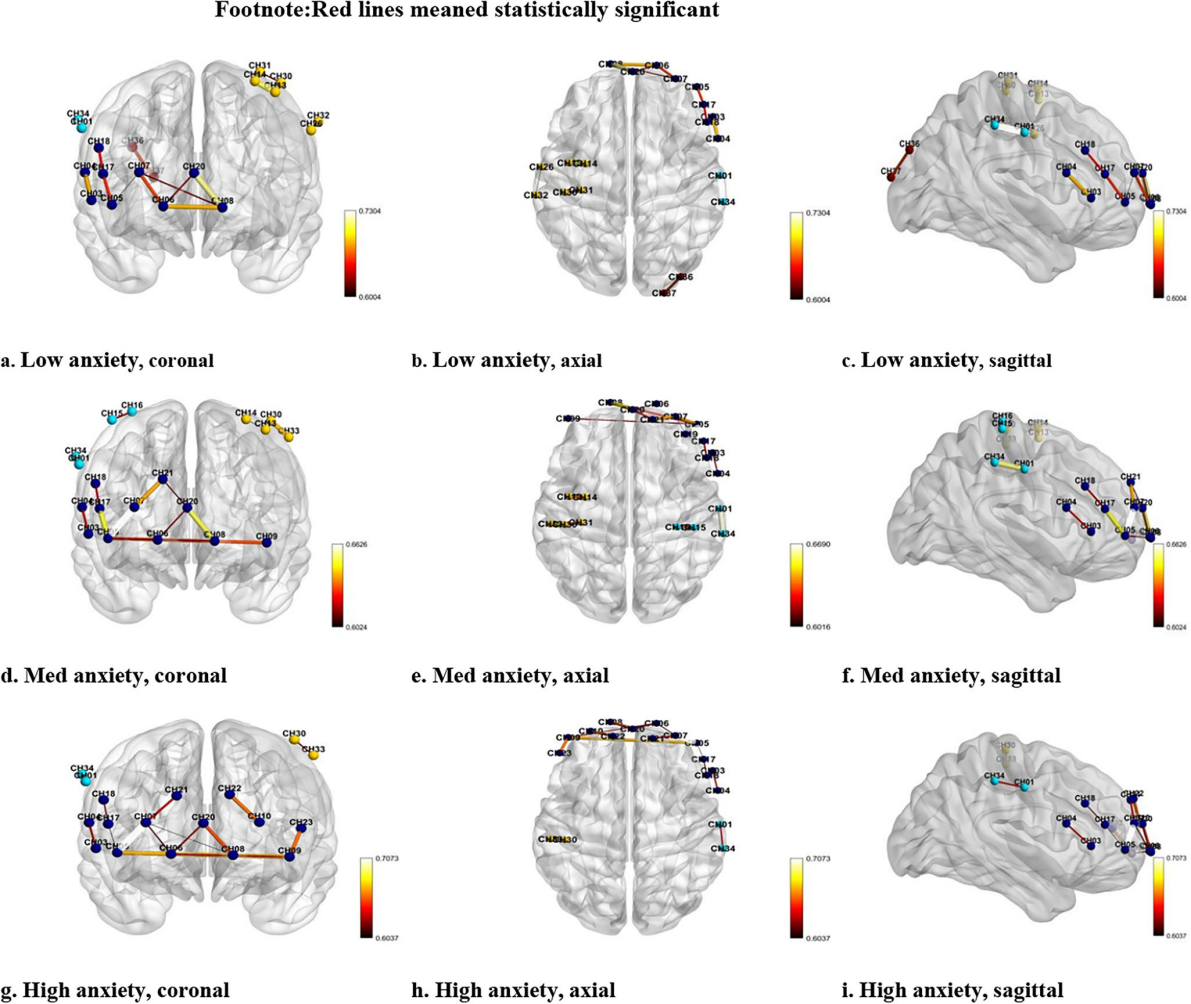
Visualization of functional connectivity during the task period: (a–c) low anxiety; (d–f) moderate anxiety; (g–i) high anxiety.

During task execution, differences in functional connectivity strength between the groups were further visualized. We found that the moderate‐ and high‐anxiety groups exhibited significantly greater functional connectivity density in the frontopolar area and dorsolateral prefrontal cortex during the task, whereas functional connectivity was relatively lower in the visual association cortex. Additionally, the high‐anxiety group showed increased functional connectivity in certain right‐side brain regions, while the moderate‐anxiety group demonstrated greater connectivity in the left‐side brain region. The threshold for connectivity strength in both groups was set at 0.58 (Figure 4).

### 3.4 Hemoglobin Oxygen (HbO) Change Between Two Groups

**FIGURE 5:**
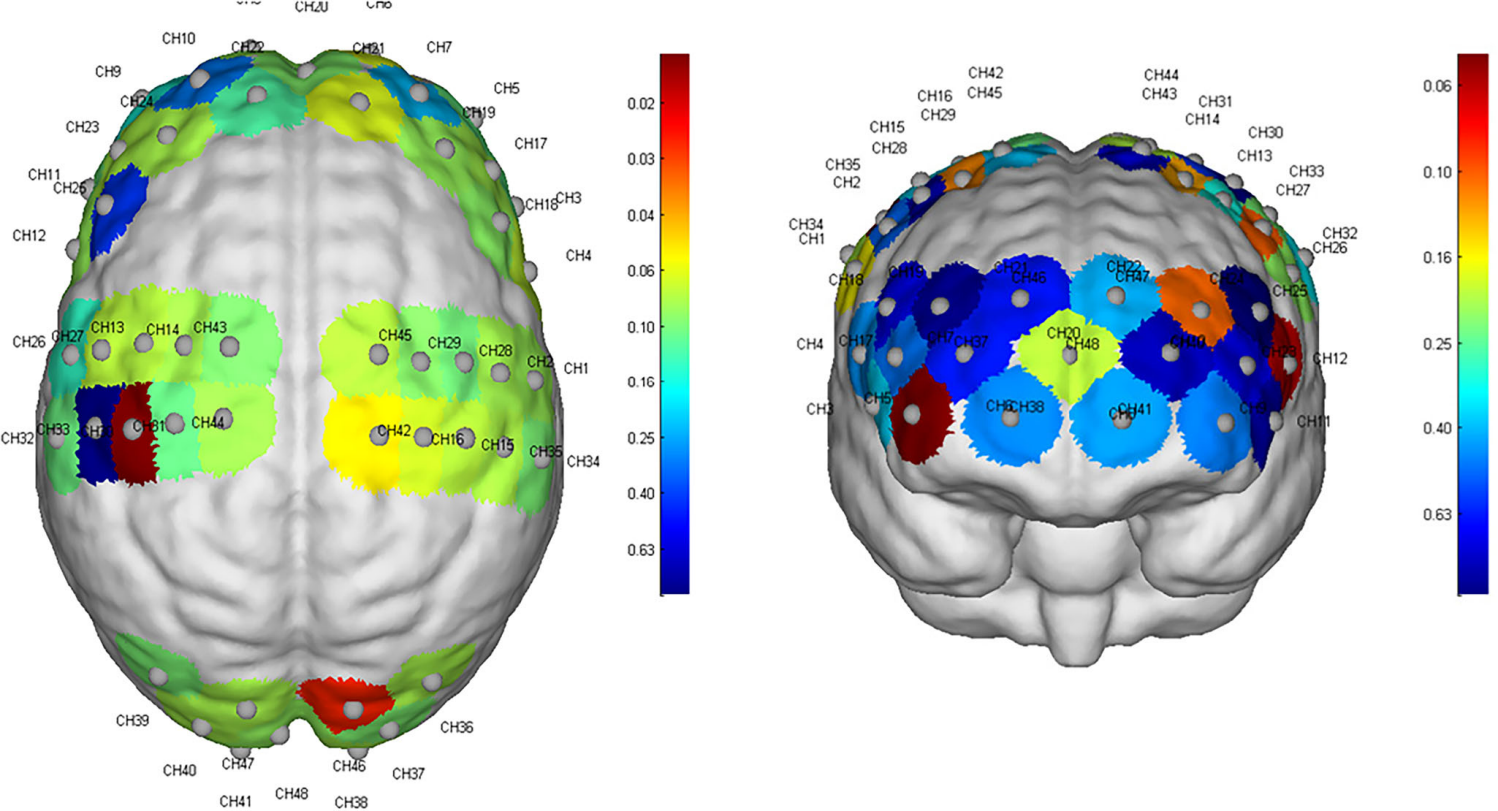
Oxygen differences between low‐anxiety group and moderate‐anxiety group. Darker red colors indicate higher levels of activation and lighter colors indicate lower levels of activation.

To compare oxyhemoglobin activation in the brain during task completion between two groups, activation maps were generated over time. In these maps, darker red colors indicate higher activation levels, while lighter colors represent lower activation levels. The average oxyhemoglobin activation in most cortical regions was significantly higher in the moderate‐ and high‐anxiety groups compared to the low‐anxiety group (Figure 5).

A two‐sample *t*‐test revealed that channels with significant differences in oxygen concentration between the moderate‐anxiety group and the low‐anxiety group were located in areas CH30, the primary somatosensory cortex (*T* = 2.6850, df = 38, *p* = 0.0107), and CH46, the visual association cortex (*T* = 2.3861, df = 38, *p* = 0.0221) (see Table 4). However, no statistically significant differences remained between the two groups after FDR correction.

**TABLE 4:** Analysis of differences in mean blood oxygen concentration indicators (low anxiety vs. moderate anxiety).

CH	Anatomic partition	*T*	Degree of freedoms	*p*
30	Primary somatosensory cortex	2.6850	38	0.0107
46	Visual association cortex (V)	2.3861	38	0.0221

### 3.5 Time‐Dependent Effects on Blood Oxygen Concentration

**FIGURE 6:**
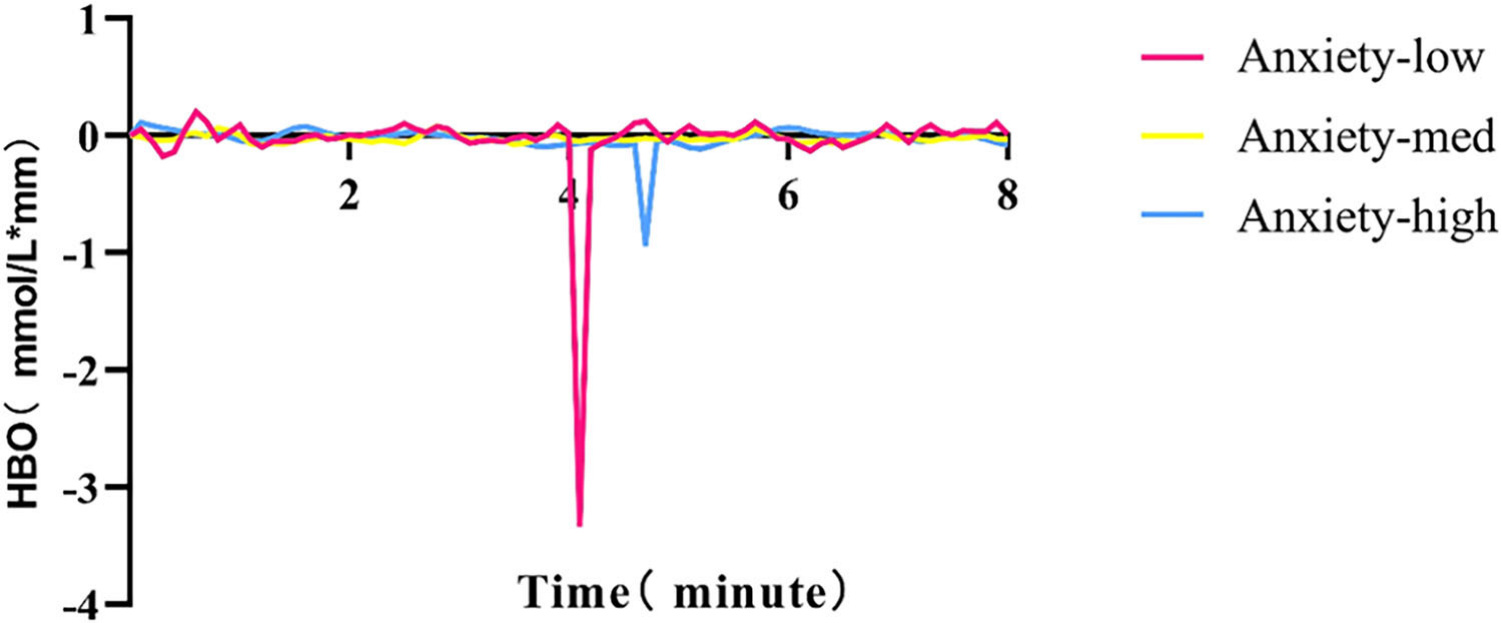
Dynamic change of blood oxygen concentration between the different groups.

**FIGURE 7:**
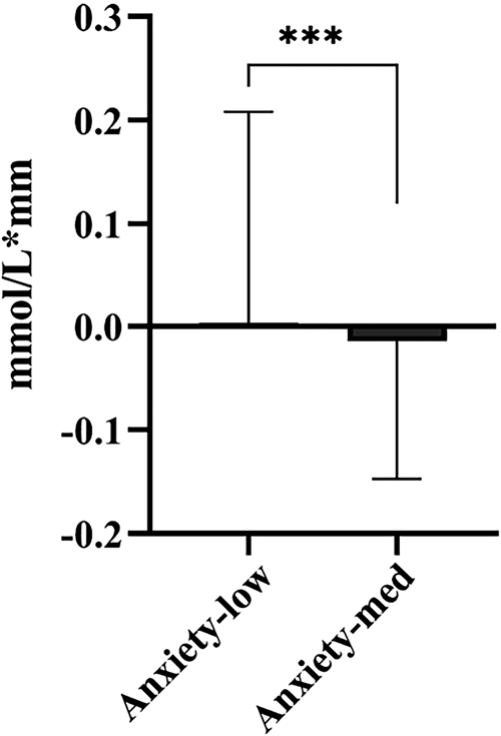
Inter‐group differences in mean HbO between low‐ and moderate‐anxiety groups (***p ≤ 0.001).

The line graph illustrates the changes in blood oxygen concentration over time among participants from different anxiety groups during the task. Notably, the moderate‐anxiety group exhibited the most stable blood oxygen levels, in contrast to the low‐anxiety group. In comparison, both the low‐ and high‐anxiety groups experienced a sharp fluctuation in blood oxygen concentration midway through the experiment (Figure 6). Additionally, a one‐way ANOVA was conducted to analyze differences in blood oxygen concentration across the groups. A significant difference was found between the mean blood oxygen concentrations of the low‐ and moderate‐anxiety groups (*p* < 0.01). See Figure 7.

### 3.6 Facial Expressions

**FIGURE 8:**
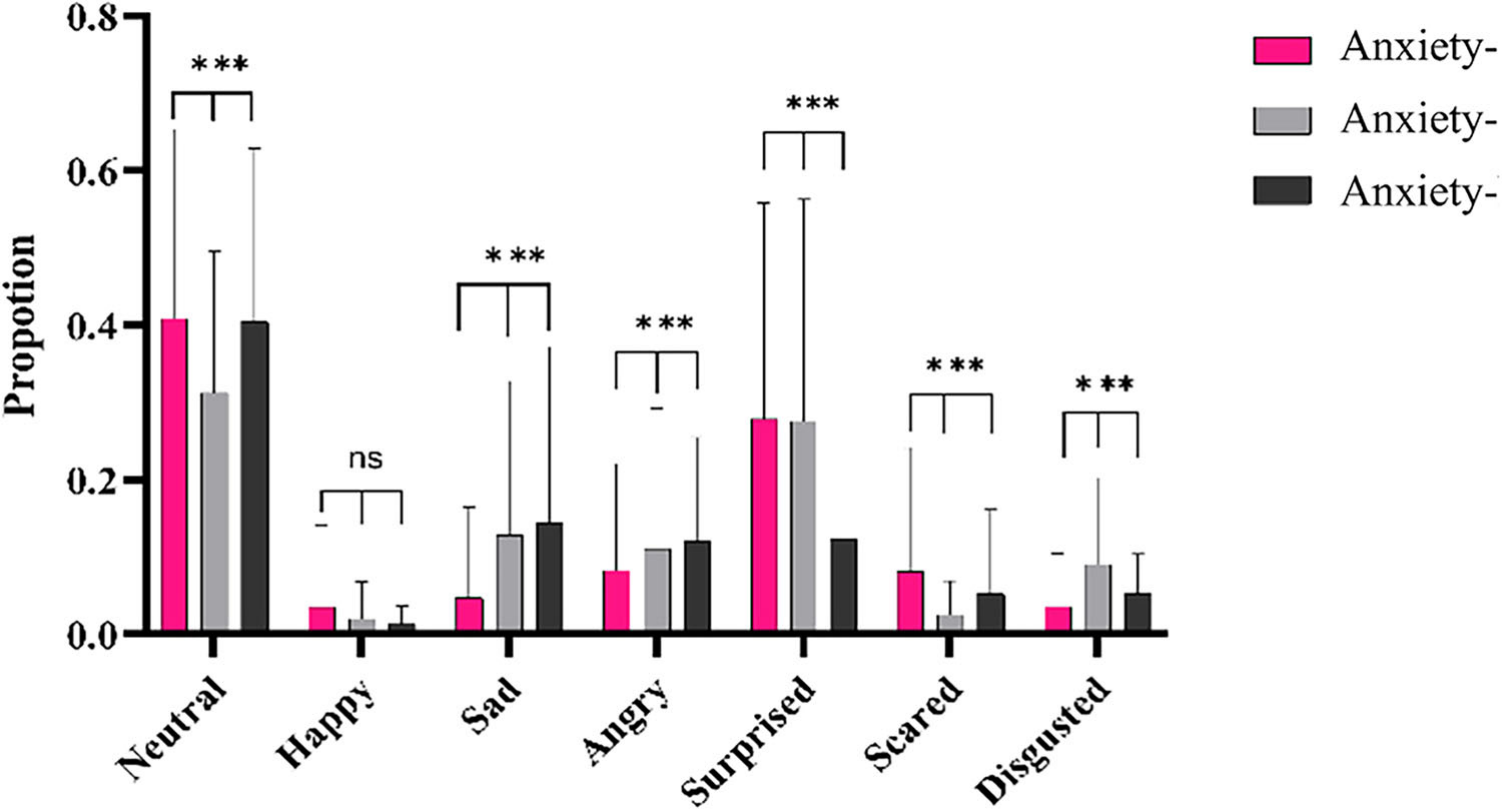
Results from facial expression analysis (***p < 0.01).

Regarding facial expressions, we found significant differences among the three groups for all emotions except “happy.” According to the results, negative emotions (sadness, anger, disgust) were significantly more pronounced in the moderate‐ and high‐anxiety groups. Interestingly, the vast majority of emotions (e.g., happy, sad, angry, surprised) in the moderate‐anxiety group were between those of the low‐ and high‐anxiety groups, and the overall range of variation in mood was minimal. See Figure 8 and Table S2.

### 3.7 Vital Signs

**FIGURE 9:**
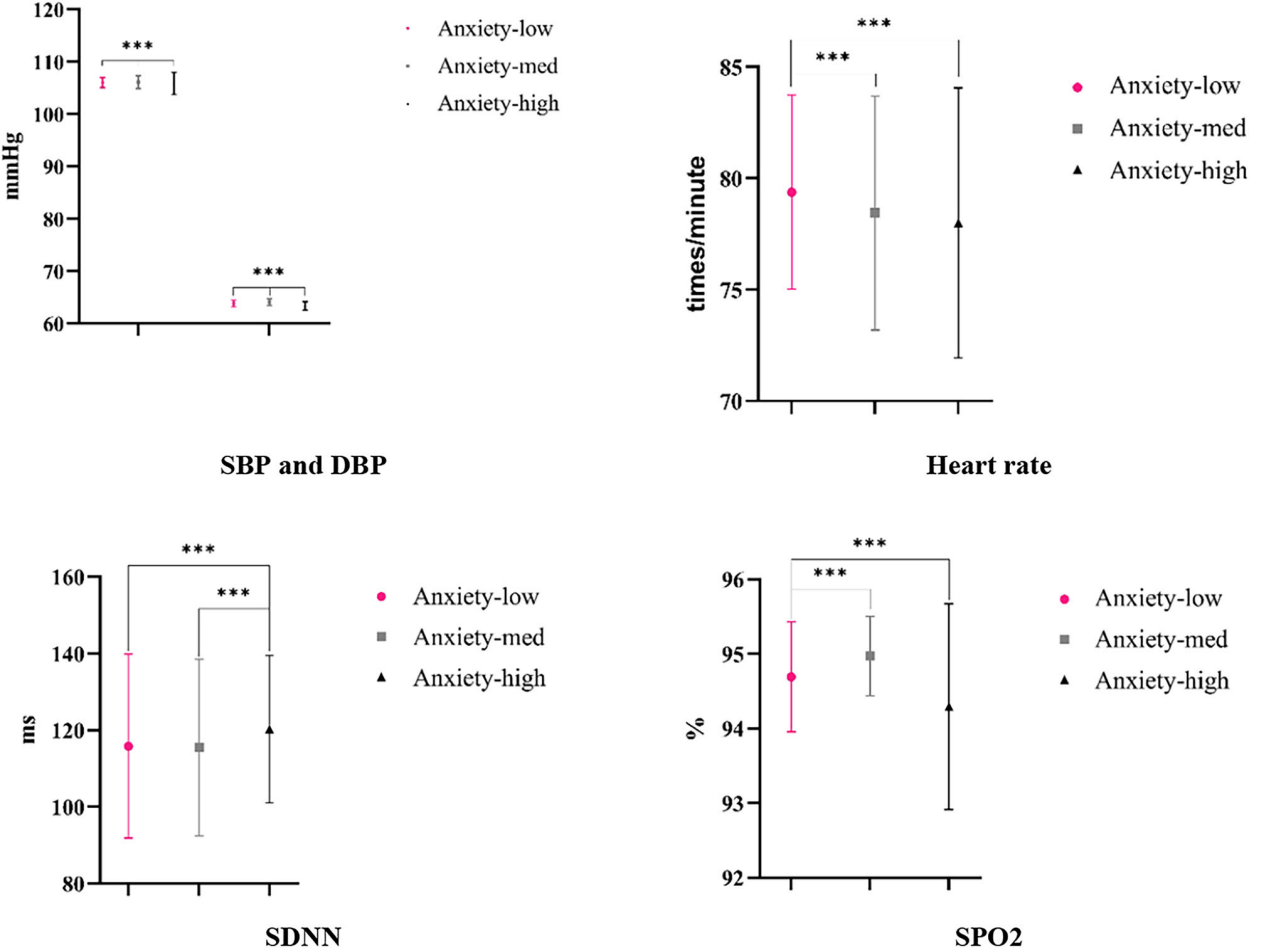
Results from vital signs analysis (***p < 0.01).

Interestingly, we found statistically significant differences in heart rate, SpO_2_, SDNN, SBP, and DBP between pairs of groups. In the high‐anxiety group, heart rate was slightly lower than in the other two groups, while SpO_2_ and heart rate exhibited the greatest fluctuations. Conversely, the low‐anxiety group had lower SDNN values than the high‐anxiety group, but the range of fluctuation was significantly greater than that of the high‐anxiety group. See Figure 9 and Table S3.

**FIGURE 10:**
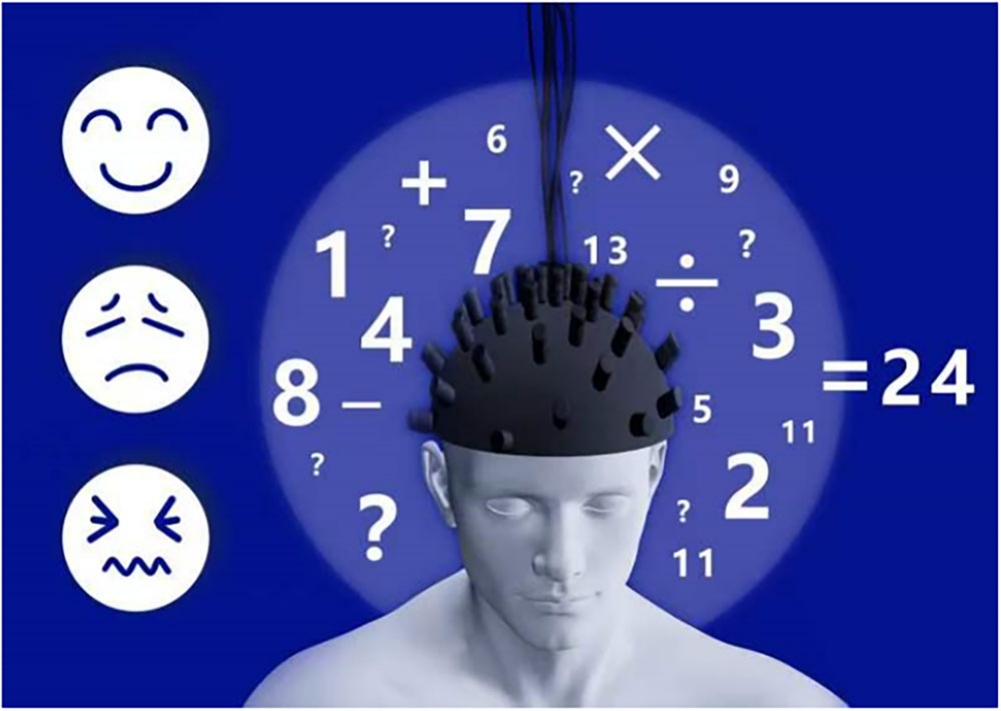
Decision‐making process under different levels of anxiety severity.

## 4 Discussion

Anxiety disorders negatively affect decision‐making processes. Investigating how varying severities of anxiety influence brain activity can enhance our understanding of the neural mechanisms underlying these disorders and provide a scientific foundation for developing more effective treatments. In this study, the posterior occipital, prefrontal, and temporal regions were engaged during the calculation task. Although between‐group variations in prefrontal functional connectivity were observed, these differences did not remain statistically significant after FDR correction. Figure 10

Our experiment has eight sections of calculations, four of which require the use of division. However, we found that people were accustomed to using addition, subtraction, and multiplication to calculate, and rarely used division. This resulted in the overall accuracy of the experiments all being low. It has been found that addition, subtraction, and multiplication usually involve lower cognitive load, while division requires more complex thinking. Therefore, when dealing with complex problems, people tend to prioritize addition, subtraction, and multiplication to minimize cognitive load (Sweller 1988). In terms of the strength of functional connectivity of brain regions between groups, note there were activation differences between the moderate‐ and high‐anxiety groups in five brain regions, including the anterior polar region, dorsolateral prefrontal cortex, supramarginal gyrus, angular gyrus, and visual association cortex. Meanwhile, both the moderate‐ and high‐anxiety groups showed strong functional connectivity, and the high‐anxiety group also showed some connectivity between right brain regions. It should be noted that these findings were observed at an uncorrected threshold and did not survive FDR correction. Based on these exploratory findings, computation is a complex skill that activates the visual, spatial, and linguistic networks, involving complex brain circuits and personalized strategies (Rapin 2016). The prefrontal cortex is responsible for higher‐order cognitive functions such as decision‐making, planning, and self‐control. Anxiety can lead to impaired functioning of the prefrontal cortex and affect decision‐making abilities (Grupe and Nitschke 2013). Studies have shown that excessive anxiety may lead to impaired prefrontal cortical functioning, which makes it difficult for patients to rationally cope with anxiety and make appropriate decisions (Mah et al. 2016). In contrast, moderate‐anxiety states have been related to moderate‐intensity prefrontal cortex activity associated with more reasonable decision‐making behavior (Helfinstein et al. 2014).

Consequently, effective cognitive functioning requires the integration of separate but interconnected cortical networks in the brain (Parks and Madden 2013). A study suggested that subjects with high anxiety were more likely to be distracted by task‐irrelevant stimulation and thus engage in more task‐irrelevant processing compared to those in a low to moderate anxiety state (Bennett and Rypma 2013). From our study, we speculatively infer that the high‐anxiety group needed more cognitive sources for decision‐making; obviously, cognitive overload may have required more attention. This situation requires more cognitive control to maintain task completion, resulting in wasted cognitive effort (Ansari and Derakshan 2011).

Regarding observed changes in blood oxygen concentration during the task, exploratory analyses indicated differences in blood oxygen levels in the regulation of brain regions between the moderate‐ and low‐anxiety groups, specifically in the primary somatosensory cortex and visual association cortex. Interestingly, the magnitude of change in blood oxygen concentration was significantly increased in the high‐anxiety group. Conversely, the moderate‐anxiety group appeared more stable, possibly indicating that their bodies were in a calmer state, as reflected in their vital signs. Also, we found significant differences between the three groups, including in heart rate, SDNN, SpO_2_, SBP, and DBP values. Interestingly, heart rate regulation in the moderate‐anxiety group was better than in the high‐ and low‐anxiety groups. The high‐anxiety group had a lower heart rate, although the fluctuation in heart rate and SpO_2_ was significant. Thus, the high‐anxiety state may overactivate the autonomic nervous system and trigger over‐responses against the resulting psychological stress, consistent with physiological indicators (Harrison et al. 2021), whereas the moderate anxiety state had the lower SDNN values and a higher range of fluctuations, which suggested an adjustment in capabilities, or otherwise an excellent performance (Thayer et al. 2012). Excessive anxiety can trigger physiological responses, which may interfere with decision‐making. These changes can heighten emotional reactions and affect rational decisions (Karver et al. 2006).

Regarding facial expressions, both the moderate‐ and low‐anxiety groups had fewer negative emotions. Although the moderate‐anxiety group showed some ambiguity, most of the emotion values were intermediate between the low‐ and high‐anxiety groups and fluctuated in the smallest range, perhaps caused by the overlapping neural circuits during attention, emotion, and decision‐making (Christakou 2014). For instance, the amygdala in patients with anxiety disorders typically shows hyperactivity, which makes patients more likely to experience excessive fear and anxiety responses to potential threats (Sah 2017). Also, excessive anxiety also affects the dopamine system in the brain, leading to altered dopamine transmission, which is associated with reward and decision processing (Hare et al. 2008). This is consistent with our hypothesis that high anxiety, conversely, increases ineffective cognition, leading to more complex and irrelevant functional connectivity in the brain, as well as more unstable physiological indicators and a more negative mood. On the other hand, most of the metrics from the moderate anxiety states appeared more moderate and stable, leading to more effective functional connectivity, smoother blood oxygenation changes, and more positive physiological metrics. With this mechanism of action, brain regions are activated significantly more efficiently, and task completion is significantly improved. In summary, the decision‐making processes in anxious states are mediated by many factors that include emotional, cognitive, and physiological responses.

Inevitably, although research has shown that decision‐making is more effective in moderate anxiety states, it faces many limitations when applied to real‐world contexts. Studies are typically conducted in controlled experimental settings that exclude external variables such as time constraints, the number of choices, and the complexity of decision‐making information. However, real‐life decision‐making often involves more complex variables and uncertainties, including emotional factors, social pressures, and multiple interactions (Barlow 2000). These complexities in real‐world scenarios may lead to differences in how anxiety affects decision‐making compared to experimental findings. Future research should focus more on the dynamic role of anxiety in complex, real‐world situations to develop more effective strategies for managing anxiety and optimizing decision‐making.

## 5 Conclusion

Moderate anxiety states can induce more effective functional connectivity and more positive and stable physiological indicators and emotional regulation to engage in decision‐making, whereas the observed patterns in functional connectivity and blood oxygenation remain exploratory and require further validation.

### 5.1 Limitation

This study involved a small number of participants and recruited only healthy adult students. Therefore, the statistical power of the study may be limited due to the small sample size, resulting in limited applicability to other age groups and populations with mental health conditions. In addition, given that normal levels of anxiety and pathological anxiety are qualitatively distinct, the generalizability of the current findings needs further examination in clinical samples. What is more, the current task, while involving decision‐making elements, also heavily recruits arithmetic processing, working memory, and executive control. Future studies should adopt well‐validated decision‐making paradigms to isolate decision processes more cleanly from other cognitive demands.

Beyond addressing these limitations, future research should also: (a) recruit larger, more diverse samples spanning different ages, educational backgrounds, and clinical statuses; (b) employ longitudinal designs to examine whether subclinical anxiety trajectories predict changes in decision‐related brain activity over time; and (c) explore other ecologically valid scenarios to better understand the transdiagnostic mechanisms linking anxiety to altered decision‐making. Such efforts will not only clarify the neurocognitive basis of anxiety‐related decision biases but also inform the development of targeted interventions for individuals with elevated anxiety symptoms.

## Author Contributions

Y.F.T., J.Y.C., and Y.X.C. collected data and conducted data analysis. Y.F.T., Y.X.C., performed the fNIRS data analysis. F.F.L. and J.Y.C. drew the pictures. M.F., F.X., and Q.H. built the idea‐concept. Y.F.T. drafted the manuscript with F.X. and Q.H. proofread this manuscript.

## Funding

This study was supported by the National Key R&D Plan (2022YFC3600500 and 2022YFC3600502), the National Natural Science Foundation of China (No. 82073833), and Key Discipline Project at the School of Public Health, Chengdu Medical College (No. 21).

## Conflicts of Interest

The authors declare no conflicts of interest.

## Supporting information




**Supplementary Tables**: brb371704‐sup‐0001‐Tables.docx

## Data Availability

Data are available upon request to the corresponding authors.
