# Supplementary material for: The Impact of Different Severities of Anxiety on Decision‐Making
Source: Brain Behav. 2026 Sep 23;16(9):e71704. doi: 10.1002/brb3.71704 (PMC13601735; doi:10.1002/brb3.71704)
Supplement: Supplementary file 1 [file BRB3-16-e71704-s001.docx]

# Supplementary File

**Table 1. The 3D MNI coordinates, anatomic partition and coverage percentage of fNIRS channels**

| Channel | MIN | | | | Anatomic partition | Percentage of Overlap（％） |
| --- | --- | --- | --- | --- | --- | --- |
|  | X | Y | Z | |  |  |
| CH01 | 63.90 | 51.74 | | 42.69 | Pre-Motor and Supplementary Motor Cortex | 0.70 |
| CH02 | 54.61 | -10.23 | | 54.99 | Pre-Motor and Supplementary Motor Cortex | 0.42 |
| CH03 | 58.57 | 32.77 | | 3.09 | Inferior prefrontal gyrus | 0.45 |
| CH04 | 62.17 | 16.12 | | 18.31 | pars triangularis Broca's area | 0.48 |
| CH05 | 47.36 | 55.72 | | 0.58 | Frontopolar area | 0.86 |
| CH06 | 19.13 | 72.12 | | -0.30 | Frontopolar area | 0.96 |
| CH07 | 32.39 | 61.89 | | 18.38 | Frontopolar area | 1.00 |
| CH08 | -13.38 | 73.21 | | -0.96 | Frontopolar area | 0.90 |
| CH09 | -43.08 | 60.31 | | -1.91 | Frontopolar area | 0.92 |
| CH10 | -27.20 | 65.58 | | 18.58 | Frontopolar area | 1.00 |
| CH11 | -57.25 | 37.36 | | -1.49 | Inferior prefrontal gyrus | 0.70 |
| CH12 | -61.17 | 21.81 | | 15.39 | pars triangularis Broca's area | 0.69 |
| CH13 | -42.37 | -2.50 | | 62.43 | Pre-Motor and Supplementary Motor Cortex | 0.99 |
| CH14 | -31.24 | -2.99 | | 68.43 | Pre-Motor and Supplementary Motor Cortex | 1.00 |
| CH15 | 45.13 | -27.19 | | 68.12 | Primary Somatosensory Cortex | 0.65 |
| CH16 | 33.71 | -26.87 | | 72.76 | Pre-Motor and Supplementary Motor Cortex | 0.31 |
| CH17 | 52.15 | 42.16 | | 17.52 | Dorsolateral prefrontal cortex | 0.86 |
| CH18 | 54.55 | 28.74 | | 31.82 | Dorsolateral prefrontal cortex | 0.96 |
| CH19 | 39.49 | 47.69 | | 31.93 | Dorsolateral prefrontal cortex | 0.69 |
| CH20 | 2.18 | 67.38 | | 17.97 | Frontopolar area | 1.00 |
| CH21 | 16.24 | 59.51 | | 34.23 | Dorsolateral prefrontal cortex | 0.62 |
| CH22 | -11.60 | 61.39 | | 35.03 | Dorsolateral prefrontal cortex | 0.56 |
| CH23 | -49.17 | 47.31 | | 14.95 | Dorsolateral prefrontal cortex | 0.77 |
| CH24 | -36.06 | 51.04 | | 30.70 | Dorsolateral prefrontal cortex | 0.5 |
| CH25 | -52.89 | 33.22 | | 30.41 | Dorsolateral prefrontal cortex | 0.97 |
| CH26 | -62.11 | -5.49 | | 41.45 | Pre-Motor and Supplementary Motor Cortex | 0.98 |
| CH27 | -54.02 | -4.11 | | 54.55 | Pre-Motor and Supplementary Motor Cortex | 0.75 |
| CH28 | 44.68 | -7.38 | | 64.03 | Pre-Motor and Supplementary Motor Cortex | 0.90 |
| CH29 | 32.84 | -7.31 | | 68.68 | Pre-Motor and Supplementary Motor Cortex | 1.00 |
| CH30 | -45.53 | -24.91 | | 67.54 | Primary Somatosensory Cortex | 0.79 |
| CH31 | -34.04 | -23.27 | | 73.41 | Pre-Motor and Supplementary Motor Cortex | 0.50 |
| CH32 | -65.98 | -27.13 | | 46.04 | Primary Somatosensory Cortex | 0.57 |
| CH33 | -55.58 | -24.46 | | 58.22 | Primary Somatosensory Cortex | 0.89 |
| CH34 | 65.82 | -32.26 | | 46.98 | Supramarginal gyrus part of Wernicke's area | 0.64 |
| CH35 | 55.54 | -29.51 | | 57.04 | Primary Somatosensory Cortex | 0.57 |
| CH36 | 36.08 | -89.67 | | 32.19 | V' | 1.00 |
| CH37 | 24.36 | -102.14 | | 15.64 | Visual Association Cortex (V) | 0.61 |
| CH38 | 15.91 | -107.38 | | 2.82 | Visual Association Cortex (V) | 0.99 |
| CH39 | -38.47 | -88.28 | | 32.52 | V' | 0.97 |
| CH40 | -26.61 | -101.37 | | 15.02 | V' | 0.54 |
| CH41 | -16.17 | -107.34 | | 0.90 | Visual Association Cortex (V) | 0.99 |
| CH42 | 21.56 | -26.57 | | 77.10 | Pre-Motor and Supplementary Motor Cortex | 0.37 |
| CH43 | -19.11 | -3.34 | | 77.18 | Pre-Motor and Supplementary Motor Cortex | 1.00 |
| CH44 | -20.76 | -22.13 | | 78.04 | Pre-Motor and Supplementary Motor Cortex | 0.59 |
| CH45 | 21.41 | -5.24 | | 76.64 | Pre-Motor and Supplementary Motor Cortex | 1.00 |
| CH46 | 14.62 | -96.96 | | 30.55 | V' | 1.00 |
| CH47 | -14.58 | -96.96 | | 30.54 | V' | 0.99 |
| CH48 | -5.63 | -103.47 | | 14.11 | Visual Association Cortex (V) | 0.99 |

**Table 2 The results of facial expression difference analysis**

| **Variable** | **Group** | **Mean** | **Std. dev** | **T** | **P** |
| --- | --- | --- | --- | --- | --- |
| Neutral | Low-Anxiety | 0.4414 | 0.3079 | -0.1353 | <0.001 |
|  | Med-Anxiety | 0.3062 | 0.2465 | -0.0237 |  |
|  | High-Anxiety | 0.4177 | 0.2604 | 0.1115 |  |
| Happy | Low-Anxiety | 0.0118 | 0.0571 | 1.4184 | <0.001 |
|  | Med-Anxiety | 1.4302 | 3.2626 | 0.0073 |  |
|  | High-Anxiety | 0.0192 | 0.0931 | -1.4111 |  |
| Sad | Low-Anxiety | 0.0592 | 0.1870 | 0.0480 | <0.001 |
|  | Med-Anxiety | 0.1072 | 0.2331 | 0.0531 |  |
|  | High-Anxiety | 0.1123 | 0.2206 | 0.0051 |  |
| Angry | Low-Anxiety | 0.0929 | 0.2368 | 0.0186 | <0.001 |
|  | Med-Anxiety | 0.1115 | 0.2406 | 0.0268 |  |
|  | High-Anxiety | 0.1196 | 0.2195 | 0.0081 |  |
| Surprised | Low-Anxiety | 0.2071 | 0.3299 | 0.0863 | <0.001 |
|  | Med-Anxiety | 0.2935 | 0.3775 | -0.0961 |  |
|  | High-Anxiety | 0.1110 | 0.2573 | -0.1824 |  |
| Scared | Low-Anxiety | 0.0751 | 0.1935 | -0.0429 | <0.001 |
|  | Med-Anxiety | 0.0322 | 0.0893 | -0.0040 |  |
|  | High-Anxiety | 0.0711 | 0.1824 | 0.0389 |  |
| Disgusted | Low-Anxiety | 0.0353 | 0.1116 | 0.0520 | <0.001 |
|  | Med-Anxiety | 0.0872 | 0.1686 | 0.0292 |  |
|  | High-Anxiety | 0.0645 | 0.1192 | -0.0227 |  |

**Table 3 The results of life signs difference analysis**

| **Variable** | **Group** | **Mean** | **Std. dev** | **T** | **P** |
| --- | --- | --- | --- | --- | --- |
| Heart-rate | Low-Anxiety | 82.0916 | 7.5787 | -1.9548 |  |
|  | Med-Anxiety | 80.1368 | 7.8562 | -2.1340 | 0.524 |
|  | High-Anxiety | 79.9577 | 7.8668 | -0.1791 |  |
| Spo2 | Low-Anxiety | 96.9843 | 0.3689 | -0.0274 |  |
|  | Med-Anxiety | 96.9569 | 0.3939 | -0.0239 | 0.903 |
|  | High-Anxiety | 96.9604 | 0.3436 | 0.0035 |  |
| SDNN | Low-Anxiety | 112.1108 | 50.9959 | 6.8415 | 0.994 |
|  | Med-Anxiety | 118.9522 | 49.7134 | 13.6282 |  |
|  | High-Anxiety | 125.7390 | 47.4704 | 6.7868 |  |
| sbp | Low-Anxiety | 108.4586 | 4.9363 | -0.2372 | <0.001 |
|  | Med-Anxiety | 108.2214 | 4.4521 | 0.5612 |  |
|  | High-Anxiety | 109.0198 | 4.4199 | 0.7984 |  |
| dbp | Low-Anxiety | 65.3738 | 2.9165 | 0.0049 | <0.001 |
|  | Med-Anxiety | 65.3787 | 2.8322 | -0.2596 |  |
|  | High-Anxiety | 65.1142 | 2.7694 | -0.2645 |  |
